# Trends in Adolescent Suicide in Hong Kong for the Period 1980 to 2003

**DOI:** 10.1100/tsw.2005.83

**Published:** 2005-09-07

**Authors:** Daniel T.L. Shek, Britta M. Lee, Joyce Chow

**Affiliations:** Social Welfare Practice and Research Centre, Department of Social Work, The Chinese University of Hong Kong, Shatin, Hong Kong

**Keywords:** Suicide, adolescence, human development, public health, trends, Hong Kong

## Abstract

This paper utilizes existing statistics on adolescent suicide to examine adolescent suicide trends and patterns in Hong Kong for the period 1980 to 2003. Several trends and patterns could be revealed from the analyses. First, there was a gradual rising trend where adolescent suicide rates in the 1990's and the early 2000's were higher than those in the 1980's. Second, suicide rates for adolescents aged 10-24 years were lower than those of other adult age groups. Third, although adolescent suicide rates in Hong Kong were lower than those reported in some English-speaking countries and Mainland China, the figures were higher than those reported in Taiwan. Fourth, suicide rates among teenagers in early adolescence were lower than those among adolescents in late adolescence. Fifth, although male adolescent suicide rates were in general higher than female adolescent suicide rates (10-24 age group), gender differences in suicide rates appeared to be moderated by age. Sixth, there was a gradual rising trend in adolescent proportional mortality rates for suicide since the 1990's. Seventh, proportional mortality rates for suicide among teenagers in early adolescence were lower than those among adolescents in late adolescence. Finally, although jumping from a height was a common method of adolescent suicide, there was a rising trend of using other methods, such as taking drugs, hanging and charcoal burning. The observed adolescent suicide phenomena are discussed in this study with reference to the socio-cultural context of Hong Kong.

## INTRODUCTION

Adolescence has been labeled as a period of "transition" in which emotional stress and instability take place[1,2]. Confronted with physical and psychological changes, adolescent development has been seen as tumultuous[3,4,5,6]. The notion that adolescence is a period of "storm and stress" appears to be supported by some research findings in which adolescents had emotional difficulties and mental health problems[7]. With reference to the various indicators of adolescent adjustment, statistics on adolescent suicide has been used as an indicator to assess adolescent turmoil and adjustment[1,2,7].

A survey of the literature shows that two measures of suicide have been used in the literature. The first measure is *suicide rate* which is calculated by dividing the total number of suicide deaths in a particular age group by the total number of persons in that age group. This rate is commonly expressed in terms of the number of suicide deaths per 100,000 persons. The second measure is *proportional mortality rate for suicide* which is calculated by dividing the total number of suicide deaths in a particular age group by the total number of deaths from all causes in that age group. This rate is commonly expressed in terms of a percentage. Although the interpretations of proportional mortality rates for suicide may be complicated, this index has been used by researchers to reflect adolescent suicide trends.

Adolescent suicide statistics in the Western contexts shows that adolescent suicide rates have increased tremendously in the post-war era[8,9,10,11,12,13]. For example, suicide rates in the 15-24 years age group in the United States rose from 5.2 in 1960 to 12.3 per 100,000 persons in 1980, which was almost a 136% increase[14]. Suicide rates for males aged 15-19 in England and Wales increased by 72% between 1970-1990[15]. Although such a rising trend has stabilized since the late 1970's, for some countries [16], such as Australia [17] and Norway[18], suicide rates have continued to climb.

## CHINESE ADOLESCENTS

Although some isolated attempts have been made to examine adolescent suicide phenomena in overseas Chinese adolescents[19,20,21], few systematic attempts have been made to examine adolescent suicide phenomena in Mainland China, Taiwan, and Hong Kong [22]. A search of the database for journal articles in PsycINFO for the past 20 years showed that few systematic reviews of trends and patterns pertinent to Chinese adolescent suicide have been carried out.

With specific reference to Hong Kong, adolescent suicide and attempted suicide have been examined in a few isolated studies[23,24,25,26,27]. For example, Yip[28] compared statistics on suicide in Hong Kong, Taiwan and Beijing and concluded that while there was a significant increase in the suicide rate among adolescents aged 15-24 years in Hong Kong in the 1980’s, the suicide rates of youth in both Taiwan and Beijing declined in the same period.

Pritchard[29] examined the patterns of suicide among adolescents aged 15-24 years in Hong Kong and the developed countries for the period between 1973-1988 and Mainland China between 1987-1988. With specific reference to gender differences in adolescent suicide, the following findings were reported:

- adolescent suicide rates in Hong Kong had dropped amongst males and remained fairly stable for females for the period under study;
- while female adolescent suicide rates for the period under study was often higher than male adolescent suicide rates in Hong Kong, the reverse was true in Japan and Western developed countries;
- while there was a significant increase in the adolescent suicide rates in nearly all Western developed countries, the suicide rates of youth in Hong Kong and Japan declined; and
- while the female adolescent suicide rate in 1988 was higher than the male adolescent suicide rate in Hong Kong and Mainland China, the reverse was true in Japan and Western developed countries.

Shek[30] examined adolescent suicide statistics in Hong Kong for the period between 1980-1991. Regarding adolescent suicide rates, several phenomena were observed: a) adolescent suicide rates in Hong Kong were relatively stable for the period under study; b) adolescent suicide rates in the 10-24 age bracket for the period under study were the lowest when compared with other adult age groups; c) adolescent suicide rates among early adolescents were lower than those among late adolescents; d) although male adolescent suicide rates were in general higher than female adolescent suicide rates (10-24 age bracket), gender differences in suicide rates appeared to be moderated by age; e) adolescent suicide rates in Hong Kong appeared to be much lower than those reported in the Western contexts; and f) adolescent suicide rates in Hong Kong appeared to be much lower than those reported in China. Shek[30] also reported several trends of adolescent suicide in Hong Kong based on proportional mortality rates for adolescent suicide.

To update our understanding of suicide trends and patterns in Hong Kong, in this paper we examine adolescent suicide in Hong Kong for the period of 1980-2003 with four specific aims in mind. First, this study examines the trend of adolescent suicide in Hong Kong in the period specified. Because the June 4$^{th}$

incident in 1989 triggered much social repercussion (such as migration and confidence crisis), adolescent suicide in the 1980's (1980 to 1989) and the 1990's to the early 2000's (1990 to 2003) will be focused upon. Secondly, this study investigates whether adolescent suicide was a serious problem in Hong Kong during this period. The answer to this question can presumably be known by analyzing the related adolescent suicide trend and comparing it with the suicide figures in other age groups and places. Thirdly, this study attempts to examine the relationship between demographic correlates (age and gender) and adolescent suicide. With reference to age, because there are findings suggesting that the mental health of adolescents deteriorates throughout the adolescent years, it will be interesting to see whether similar trends occur for adolescent suicide. Regarding gender, because previous findings showed that there were atypical gender differences in suicide rates in Hong Kong adolescents [29,30] and that gender differences in proportional mortality rates have not been properly examined in the literature, gender differences in different adolescent age groups in terms of suicide rates and proportional mortality rates will be examined in this paper. Finally, methods of adolescent suicide in Hong Kong will be investigated to see whether there are any distinct patterns involved.

## METHODS

Based on the statistics compiled by the Census and Statistics Department of the Government of the Hong Kong Special Administrative Region, information on: a) the total number of suicide deaths for different age groups in the total, male and female populations; b) the total number of deaths from all causes for different age groups in the total, male and female populations; and c) the total number of people for different age groups in the total, male and female populations, in the period from 1980 to 2003 was collected. Based on the above information, relevant suicide rates and proportional mortality rates for suicides were computed.

As far as the definition of adolescence is concerned, adolescence is defined as the period between age 10 to age 24 years, with the 10-14, 15-19 and 20-24 age brackets representing early adolescence, middle adolescence, and late adolescence respectively. This conception is a commonly adopted one and it fits nicely into the age brackets employed by the Census and Statistics Department in Hong Kong. However, because different age brackets have been used by different authors[13,31,32,33,34,35,36,37,38,39], adolescent suicide rates and proportional mortality rates for suicide based on some of these age brackets are also presented in this paper for comparison purposes. In addition, relevant statistics from the World Health Organization[32,33,34,35,36,37,38,39] and governments in the Asian and Western contexts were also included for comparisons (see Tables 2,3,4,6 and 7). Finally, with specific reference to the methods of adolescent suicide, relevant statistics were obtained from the Coroners' Office for the period of 1989 to 2003[40,41,42,43,44,45,46,47,48,49,50,51,52,53,54].

## RESULTS

The suicide rates in different adolescent age groups for the different populations in the period from 1980 to 2003 are presented in Table 1. With specific reference to suicide rates for adolescents aged 10 to 24 years, several phenomena were observed. First, although there was no abrupt change in adolescent suicide rates (10-24 ages group) for the period under study, there was a gradual rising trend in the period of 1990-2003. The suicide rates were highest in the early 2000's (mean=7.17 for 2000 to 2003), which was followed by those in the 1990's (mean=6.02 for 1990 to 1999) and the 1980's (mean=4.40 for 1980 to 1989). Second, results showed that suicide rates for adolescents aged 10-24 years were generally lower than those of the adult age groups (Figure 1).

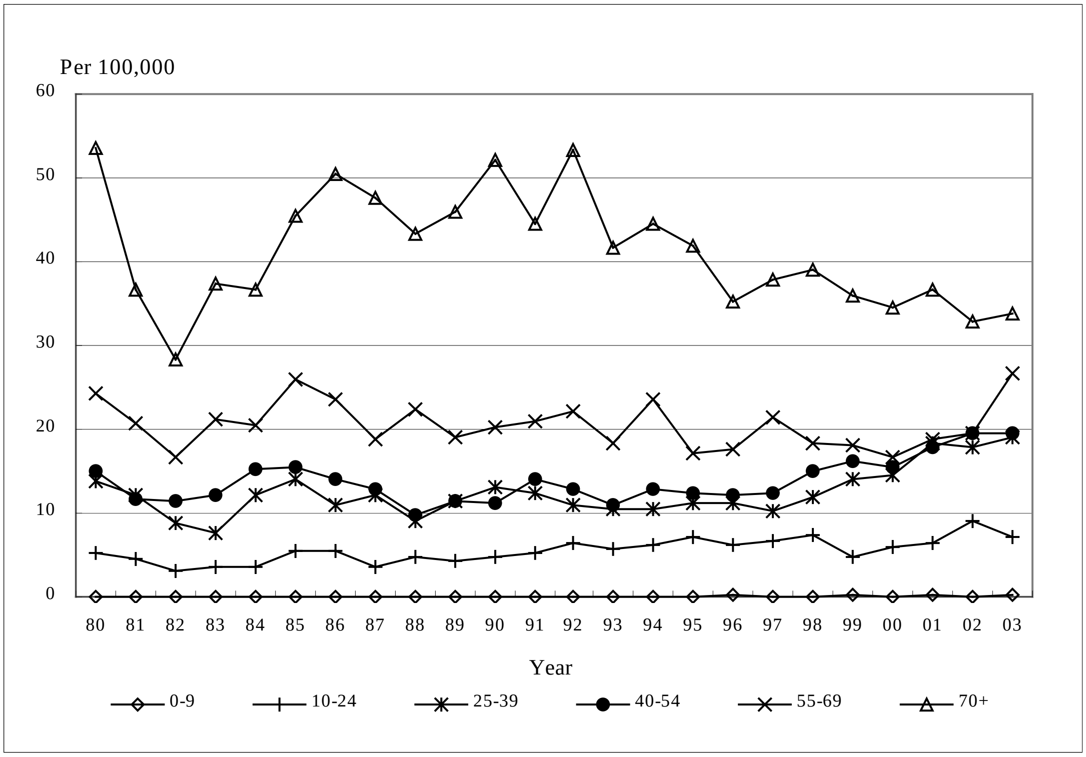

**FIGURE 1.** Suicide rates by age groups for the total population.

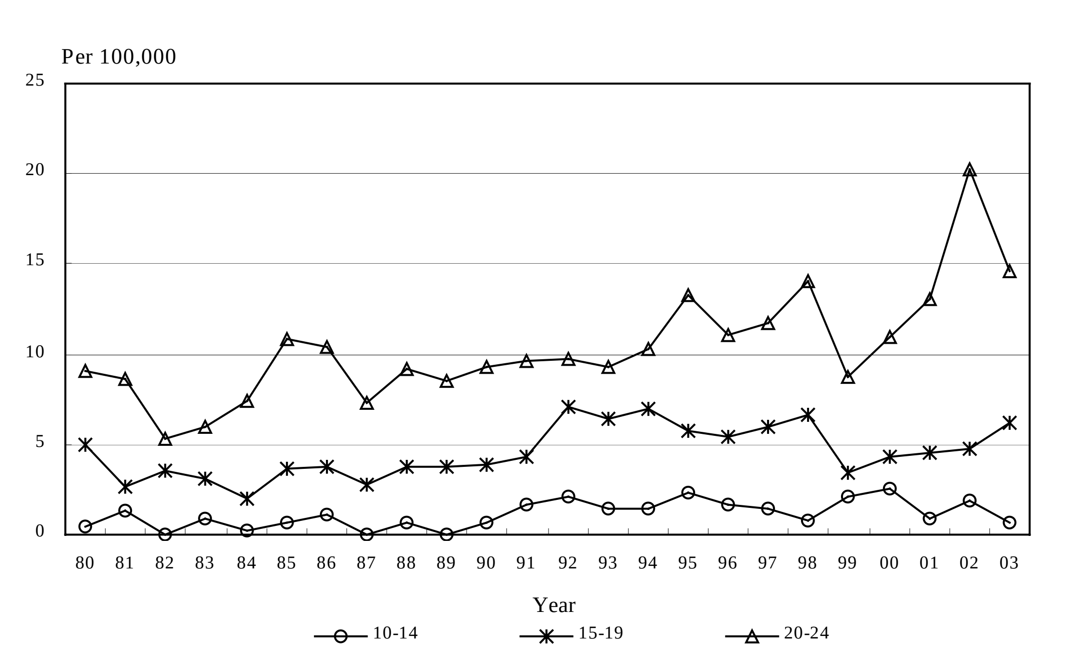

**FIGURE 2.** Suicide rates in different adolescent age groups for the total population.

Third, when the suicide rates in Hong Kong were compared with the figures in some Western countries and Chinese communities, it was found that adolescent suicide rates in Hong Kong appeared to be lower than most of the English-speaking countries, although the gap was narrowing. In addition, although adolescent suicide rates in Hong Kong appeared to be lower than those reported in Mainland China, they were higher than those of Taiwan. The findings on these comparisons are presented in Tables 2, 3 and 4.

Fourth, concerning the relationship between age and adolescent suicide, the data in Table 1 generally showed that suicide rates were highest in late adolescence, which was followed by middle adolescence and early adolescence and this pattern held for the total, male and female populations. The suicide rates for different adolescent age groups in the total population are graphically presented in Figure 2.

Finally, regarding gender differences in adolescent suicide rates, although male adolescent suicide rates were in general higher than female adolescent suicide rates in the 10-24 age bracket, gender differences in suicide rates appeared to be moderated by age. While female suicide rates were higher than male suicide rates in early adolescence (10-14 age bracket), the reverse was true for late adolescence (20-24 age bracket) and the pattern of female and male suicide rates was mixed in middle adolescence (15-19 age bracket). The suicide rates for different adolescent age groups by gender are graphically presented in Figure 3.

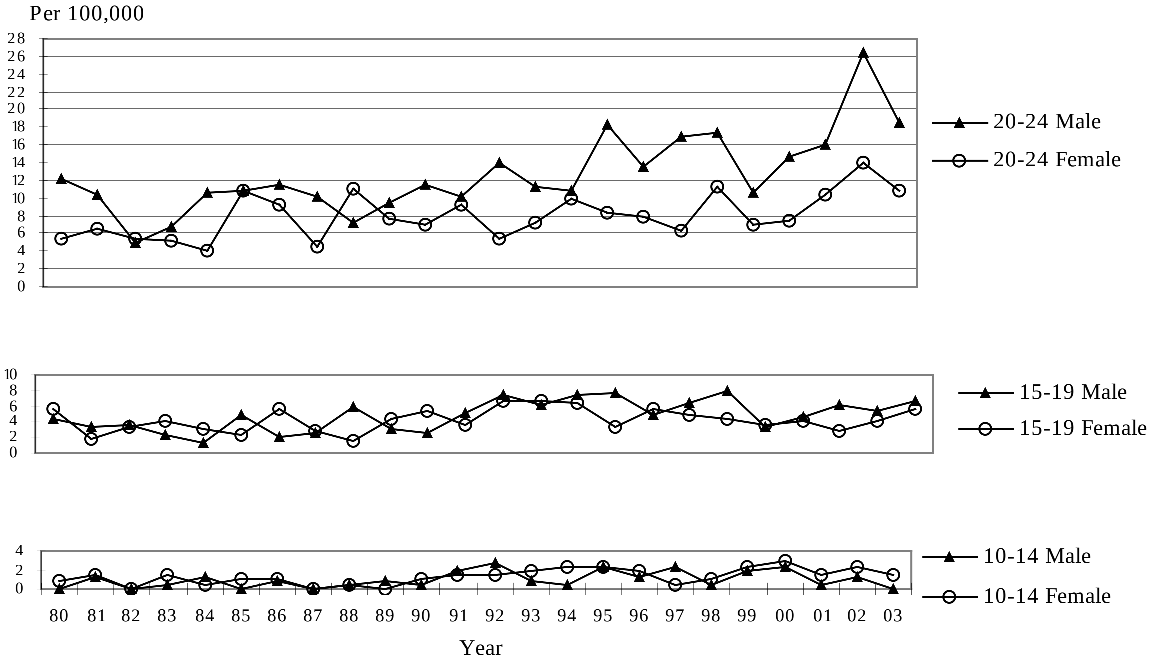

**FIGURE 3.** Suicide rates in different adolescent age groups by gender.

## PROPORTIONAL MORTALITY RATES FOR SUICIDE

The proportional mortality rates for suicide in different age groups for the different populations in the period between 1980 and 2003 are presented in Table 5. Several phenomena can be highlighted from the data. First, with reference to the total, male and female populations, proportional mortality rates for suicide in Hong Kong adolescents in the past two decades fluctuated, ranging from less than 10% to over

30%. Second, as compared to other age groups, proportional mortality rates in the 10-24 age bracket in the past two decades were roughly the second highest rates in the total population (Figure 4).

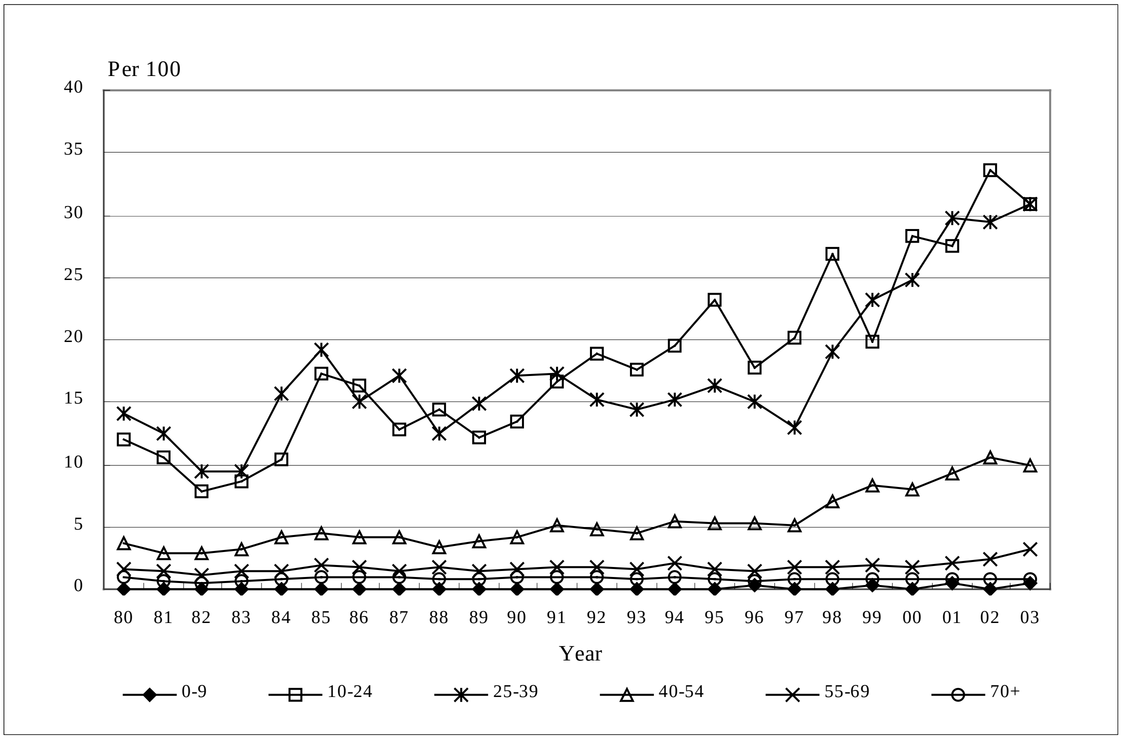

**FIGURE 4.** Proportional mortality rates for suicide for the total population.

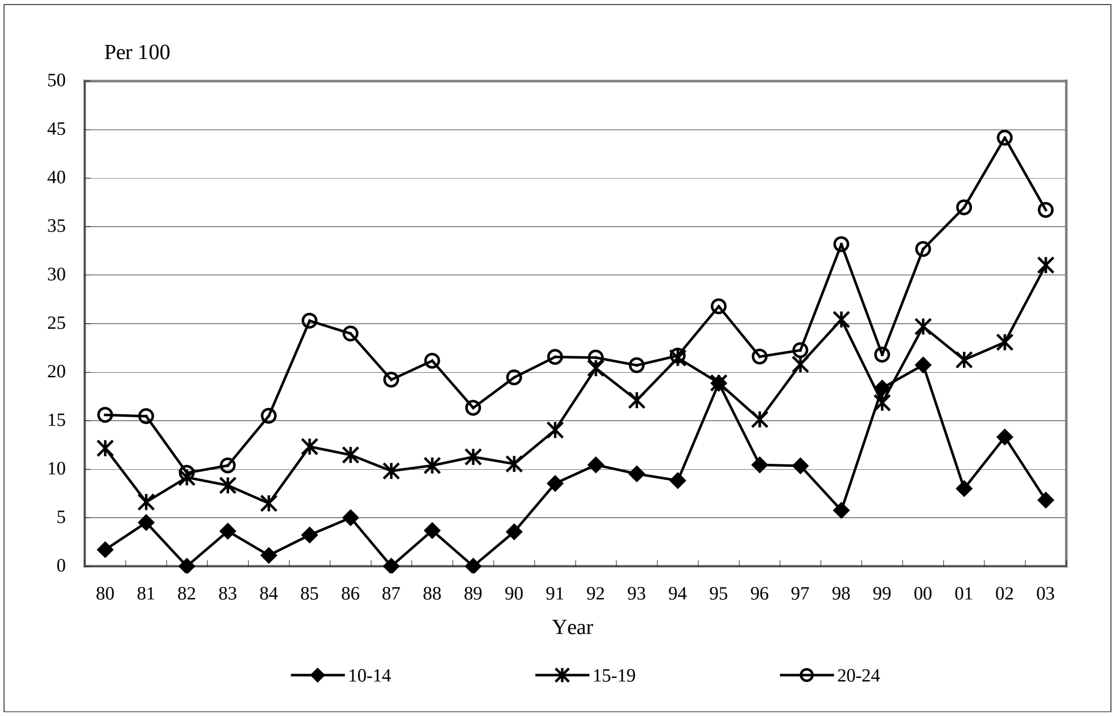

**FIGURE 5.** Proportional mortality rates for suicide in different adolescent age groups for the total population.

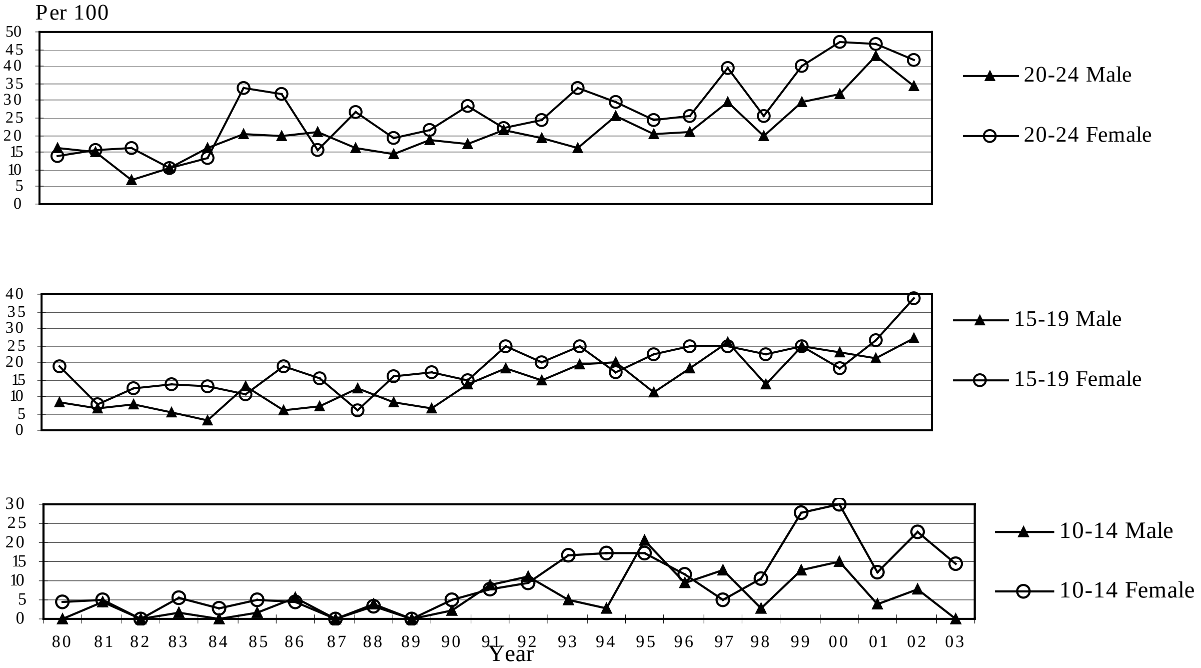

**FIGURE 6.** Proportional mortality rates for suicide in different adolescent age groups by gender.

Third, when compared with other places, proportional mortality rates for suicide in Hong Kong appeared to be roughly similar to those reported in the Asian contexts, but lower than those reported in Mainland China (see Table 6 and 7). While the proportional morality rates in Hong Kong were similar to those reported in the Western contexts in the 1980's, relatively higher proportional mortality rates as compared to some English-speaking countries in the 1990's and the early 2000's were observed.

Finally, similar to adolescent suicide rates, proportional mortality rates for suicide were highest in late adolescence, which was followed by middle adolescence and early adolescence (Figure 5). However, in contrast to suicide rates, female proportional mortality rates for suicide were generally higher than male proportional mortality rates for suicide (Figure 6).

## METHODS OF SUICIDE

Tables 8, 9 and 10 show that amongst the different methods of suicide, jumping from a height was the most common method used in adolescent suicide and this form of suicide appeared to be more frequent among female than male adolescent suicide. However, there were also signs suggesting that other methods of suicide, such as taking drugs, hanging and charcoal burning, became more popular since the 1990's. Of all the suicide methods used for the total population, the rate of charcoal burning has increased from 3% in 1998 to 26.4% in 2003, replacing the traditional method of hanging as the second most common method of suicide[55]. Unfortunately, because the age brackets adopted by the Coroners' Office are not refined enough, it is difficult to examine methods of suicide in the different stages of adolescence.

## DISCUSSION

The purpose was to study phenomena pertinent to adolescent suicide based on analyses of suicide statistics in Hong Kong for the period of 1980 to 2003. Four basic questions concerning the trend, severity, relationship between demographic factors (age and gender) and adolescent suicide, and methods of adolescent suicide were addressed.

Concerning the trend of adolescent suicide in the period under study, the suicide rates displayed in Table 1 show that although there was no abrupt fluctuation in adolescent suicide rates (10-24 ages group) for the period under study, there was a gradual rising trend in the 1990’s and the early 2000’s as compared to the 1980’s. A similar rising trend was observed when proportional mortality rates for adolescent suicide were under focus (Table 5).

How can we account for the gradual rise in adolescent suicide rates in Hong Kong since the 1990’s? Consistent with the experiences of Western societies[56], this phenomenon can probably be explained in terms of the worsening of the economic situation of Hong Kong in the latter half of the 1990’s. This conjecture is in line with the thesis that economic stress and social changes have an intimate link with adolescent psychological well-being and adjustment.

According to socio-ecological theories, adolescent adjustment is closely related to social changes. With reference to the social changes in Hong Kong in the last two decades, there were several changes that might have adversely affected the adjustment of adolescents. First, because of uncertainty about the future, anticipation of the 1997 handover to China created a confidence crisis. The confidence crisis intensified after the June 4$^{th}$ incident in 1989 that triggered the migration trend. Second, because inflation and property prices were notoriously high before the handover, the bubble economy created much economic strain for families in Hong Kong in the early to mid 1990’s. Third, the economic situation declined sharply after the handover. The Asian financial crisis in 1997 destroyed the stable lives and shattered the dreams of people in Hong Kong. Many people were in debt and even became bankrupt because of the sharp fall in the value of the property, downturn in the industries, and unemployment. People in Hong Kong felt hopeless in such an unpleasant social and political atmosphere. Finally, because of the declining economic conditions, adolescents had more difficulty in finding jobs and youth unemployment had increased sharply. In short, the above-mentioned social changes in the 1990’s created much stress for people in Hong Kong, especially adolescents and their families.

Even though there has been a gradual rise in adolescent suicide rates and proportional mortality rates since the 1990’s, is the problem a serious one? With respect to the question of whether adolescent suicide in Hong Kong in the period under study was a “serious” problem, findings based on adolescent suicide rates revealed two points. First, adolescent suicide rates were lower than suicide rates in other adult age groups (see Figure 1). Second, the related rates were lower than those reported in most of the English-speaking countries (but the gap was narrowing) and Mainland China, although suicide rates in Hong Kong were higher than those in Taiwan (see Tables 2, 3 and 4). Based on these two observations, it can be concluded that adolescent suicide expressed in terms of suicide rates is not particularly serious for the period of 1980 to 2003, but there is a worsening trend.

On the other hand, findings based on the proportional mortality rates (Table 5 and Figure 4) showed that proportional mortality rates for adolescent suicide were the second highest rates amongst all age groups and such rates appeared to be higher than some Western countries in some of the years under review (Tables 6 and 7). These findings suggest that adolescent suicide might be an emerging social problem in Hong Kong. However, it is noteworthy that interpretations of the findings based on proportional mortality rates for adolescent suicide should be made with caution, because other extraneous variables might have affected the related rates. For example, while medical advances will tend to inflate the proportional mortality rate for suicide, increased occurrence of other causes of death will tend to deflate the rate. Conservatively speaking, the present findings based on adolescent suicide rates generally suggest that the problem in adolescents was relatively not serious as compared to other age groups and other places.

It would be interesting to ask why adolescent suicide rates in Hong Kong were relatively not serious as compared to other age groups and other places. There are at least two protective factors that may help to reduce adolescent suicide rates in Hong Kong. The first protective factor is related to some of the traditional Chinese beliefs that help to inhibit suicide behavior. For example, killing or hurting oneself is regarded as an unfilial act in the Confucian teachings and suicide is not encouraged in the Buddhist and Taoist thoughts. In addition, traditional Chinese beliefs, particularly those related to Confucian thoughts, tend to cultivate the virtues of patience and endurance that might prevent one from killing himself so easily.

The second protective factor is related to the pragmatic cultural values in Hong Kong. For example, the popular beliefs that "it is wise not to take things too hard" and "if you can keep a mountain (i.e., keep one's life and well-being), you don't have to worry that there is not enough log (i.e., opportunities and chances)" can provide a buffer against suicide.

One important theoretical implication of the present findings is that they do not seem to concur with the "tumultuous" portrait of adolescence. If adolescent suicide rates are used as the sole indicator of adolescent mental health, the present findings suggest that the mental health of adolescents in Hong Kong is relatively good, although there is a gradual rising trend. However, when the results of other epidemiological studies on adolescent mental health in Hong Kong[57,58,59] are also taken into account, the picture becomes more complicated for such studies generally point out that roughly 20% to 30% of the adolescents under study could be identified as "probable" psychiatric cases. In other words, the existing literature suggests that although quite a significant proportion of adolescents might be "at-risk" in their mental health, only a very small proportion of adolescents were actual suicidal cases. Perhaps further studies should be carried out to clarify the possible links between psychiatric morbidity and suicide in adolescents in Hong Kong.

The present findings also suggest that social and economic changes are important factors related to adolescent suicide. In his interpretation of the relatively low adolescent suicide rates in Hong Kong in the 1980’s, Shek [30] suggested that the “abundance of economic opportunities” could explain the relatively low level of adolescent suicide in Hong Kong. In the same light, development of the bubble economy in early to mid 1990’s and the lack of economic opportunities in late 1990’s might contribute to the rise in adolescent suicide rates since the 1990’s. Because few studies have been conducted to examine the relationship between economic factors and adolescent development, further studies along this line should be conducted.

With reference to the relationship between age and adolescent suicide, data based on both adolescent suicide rates and proportional mortality rates for suicide suggest that adolescent suicide accelerates with the increase of age. This observation is consistent with the findings of Shek[57,58] that psychiatric morbidity climbs with an increase in age. This finding also mirrors the observation that perceived family relations deteriorate throughout the adolescent years. The present findings suggest that some preventive measures of adolescent suicide could possibly be done at an earlier stage.

Regarding gender differences in adolescent suicide, while the observed adolescent suicide rates were generally consistent with the general picture that adolescent suicide is more prevalent in males than females, the data also suggest that gender differences in suicide might be moderated by age. While female suicide rates were higher than male suicide rates among adolescents aged 10-14, the reverse was true among adolescents aged 20-24. Among adolescents aged 15-19 years, the pattern of female suicide rates and male suicide rates was mixed. While this observation is consistent with Pritchard’s[29] observation of atypical gender differences in suicide rates among adolescents aged 15-24 years in Hong Kong, the present findings suggest that the atypical pattern was confined to the period of early adolescence only. Similarly, the present findings echoed those reported by Pritchard[60] that suicide rates amongst young women were higher than those of young men in the 15-24 age group in China, but the opposite pattern was found in Japan and Western countries[29,61]. Because Pritchard’s study[60] was not based on suicide statistics over a period of time, the generalizability of the findings may be limited. In contrast, the present study generates trend data on gender differences in suicide rates in Hong Kong that can provide a more stable picture on this issue. Finally, the present study suggests different age brackets should be used when gender differences in suicide statistics are examined.

When proportional mortality rates for suicide are under focus, the observed pattern is consistent with the previous findings that females displayed more psychiatric morbidity than males[62]. Because gender differences in proportional mortality rates for suicide are grossly under-researched in the literature[60,61], the present findings are interesting additions to the litertaure.

How can we account for the above gender differences in suicide rates and proportional mortality rates? Pritchard[60] suggested that the higher reported suicide rates among Chinese females might be due either to under-reporting for male suicides in a male-dominated society (such as the fear of losing face), or to the lower social status and self-esteem of Chinese women. Because Hong Kong is quite a Westernized society and the development of forensic medicine is on par with the international standard, the possibility that the relatively higher female suicide rates are due to under-reporting is not high. In fact, the relatively higher female suicide rates in younger adolescence and higher proportional mortality rates in the 10-24 age bracket may suggest the operation of cultural factors, such as gender inequalities in terms of social opportunities and expectations in the Chinese culture, that contribute to the relatively poorer mental health amongst Chinese women[62].

Finally, concerning the methods involved in adolescent suicide, one obvious pattern that could be observed from the data is that jumping from a height was a commonly used suicide method and it appeared that it was more favored by female adolescents (Tables 8, 9 and 10). There are several characteristics of "jumping from a height" as a method of suicide[30]. First, it is lethal and irrevocable. Second, the dying process is normally quick. Third, it often attracts public and media attention. This observed pattern appeared to be inconsistent with the adolescent suicidal phenomena reported elsewhere[10,16]. The predominant use of "jumping from a height" as a suicidal method could possibly be due to the abundance of high-rise buildings in Hong Kong and the non-availability of other methods, such as firearms.

While jumping from a height was the most common method of suicide in the past two decades, there was an increase in the use of other methods of suicide, including taking drugs, hanging and charcoal burning (Tables 8, 9 and 10). The rapid increase of the use of charcoal burning to commit suicide and the use of a wide range of suicide methods by Hong Kong adolescents in recent years suggest that suicide prevention methods should be more diversified. For example, in the case of using charcoal burning as a method of suicide, printing optimistic slogans and telephone numbers of suicide prevention centers on the plastic bags containing charcoal would be helpful. Based on evidence and research, the restriction of charcoal sales to near barbecue sites would be an effective method to help prevent suicide[63]. The implementation of drug education programs in the school context would also help students understand the dangerous aspects of abusing drugs. In addition, encouraging the mass media in responsible reporting would help prevent imitation effects of suicide cases and engaging them in a positive manner would be beneficial in promoting suicide prevention programs[64].

## CONCLUSION

Although the present analyses showed that the problem of adolescent suicide in Hong Kong from 1980 to 2003 was relatively not serious as compared to other adult age groups and other places, both suicide rates and proportional mortality rates showed gradual rising trends in the past two decades. Some interesting gender differences in adolescent suicide rates and proportional mortality rates were also observed. With the intensification of economic re-structuring in Hong Kong, it is suggested that suicide prevention programs should be implemented in the early adolescent years.

## ACKNOWLEDGEMENT

This work was financially supported by the Research Grants Council of the Government of the Hong Kong Special Administrative Region, Hong Kong (Grant CUHK4293/03H) and Wofoo Foundation.

## BIOSKETCHES

**Daniel T.L. Shek, PhD, FHKPs.S, BBS, JP**, is professor at the Department of Social Work, the Chinese University of Hong Kong. He is on the editorial board of several scientific journals and has published extensively in international journals on social work, Chinese and English language, mental health, well-being, quality of life, adolescence, disadvantaged families and drug abuse. E-mail: DANIELSHEK@CUHK.EDU.HK. Website: HOME PAGE: http://www.cuhk.edu.hk/swk

**Britta M. Lee,** MDes, Social Welfare Practice and Research Centre, Department of Social Work, The Chinese University of Hong Kong, Hong Kong. E-mail: bmlee@swk.cuhk.edu.hk

**Joyce T.W. Chow**, BSSC, RSW, Social Welfare Practice and Research Centre, Department of Social Work, The Chinese University of Hong Kong, Hong Kong. E-mail: twchow@cuhk.edu.hk

## TABLE 1

### Adolescent suicide rates in Hong Kong (1980-2003)

| Age group | | 1980 | 1981 | 1982 | 1983 | 1984 | 1985 | 1986 | 1987 | 1988 | 1989 | 1990 | 1991 |
|---|---|---|---|---|---|---|---|---|---|---|---|---|---|
| 10-14 | *T | 0.44 | 1.35 | 0.00 | 0.92 | 0.23 | 0.69 | 1.14 | 0.00 | 0.71 | 0.00 | 0.71 | 1.67 |
| | *M | 0.00 | 1.31 | 0.00 | 0.44 | 1.31 | 0.00 | 0.90 | 0.00 | 0.46 | 0.92 | 0.45 | 1.83 |
| | *F | 0.90 | 1.39 | 0.00 | 1.43 | 0.48 | 0.96 | 0.96 | 0.00 | 0.49 | 0.00 | 0.98 | 1.49 |
| 15-19 | T | 5.00 | 2.64 | 3.50 | 3.10 | 2.04 | 3.62 | 3.74 | 2.73 | 3.72 | 3.74 | 3.92 | 4.31 |
| | M | 4.35 | 3.40 | 3.58 | 2.26 | 1.18 | 4.92 | 2.11 | 2.62 | 5.79 | 3.14 | 2.65 | 5.06 |
| | F | 5.68 | 1.82 | 3.42 | 4.00 | 2.95 | 2.21 | 5.52 | 2.86 | 1.46 | 4.38 | 5.29 | 3.50 |
| 20-24 | T | 9.05 | 8.64 | 5.27 | 5.96 | 7.43 | 10.88 | 10.40 | 7.34 | 9.13 | 8.51 | 9.31 | 9.67 |
| | M | 12.26 | 10.45 | 5.06 | 6.74 | 10.54 | 10.83 | 11.42 | 10.10 | 7.28 | 9.41 | 11.49 | 10.11 |
| | F | 5.49 | 6.64 | 5.49 | 5.13 | 4.15 | 10.93 | 9.35 | 4.48 | 11.05 | 7.58 | 7.05 | 9.23 |
| 10-24 | T | 5.14 | 4.52 | 3.21 | 3.60 | 3.62 | 5.58 | 5.55 | 3.67 | 4.82 | 4.29 | 4.77 | 5.34 |
| | M | 5.97 | 5.48 | 3.16 | 3.48 | 4.46 | 5.86 | 5.43 | 4.64 | 4.81 | 4.38 | 4.98 | 5.73 |
| | F | 4.24 | 3.47 | 3.26 | 3.72 | 2.72 | 5.29 | 5.68 | 2.63 | 4.83 | 4.20 | 4.54 | 4.93 |

| Age group | | 1992 | 1993 | 1994 | 1995 | 1996 | 1997 | 1998 | 1999 | 2000 | 2001 | 2002 | 2003 |
|---|---|---|---|---|---|---|---|---|---|---|---|---|---|
| 10-14 | T | 2.13 | 1.41 | 1.40 | 2.31 | 1.60 | 1.41 | 0.71 | 2.09 | 2.55 | 0.93 | 1.84 | 0.69 |
| | M | 2.75 | 0.92 | 0.45 | 2.25 | 1.33 | 2.29 | 0.46 | 1.81 | 2.25 | 0.45 | 1.34 | 0.00 |
| | F | 1.47 | 1.93 | 2.39 | 2.38 | 1.89 | 0.49 | 0.98 | 2.39 | 2.86 | 1.44 | 2.37 | 1.42 |
| 15-19 | T | 7.10 | 6.40 | 6.99 | 5.70 | 5.16 | 5.68 | 6.23 | 3.46 | 4.63 | 4.48 | 4.77 | 6.20 |
| | M | 7.52 | 6.20 | 7.43 | 7.78 | 4.80 | 6.43 | 8.01 | 3.38 | 4.65 | 6.10 | 5.30 | 6.71 |
| | F | 6.64 | 6.62 | 6.52 | 3.45 | 5.55 | 4.90 | 4.39 | 3.54 | 4.04 | 2.77 | 4.21 | 5.66 |
| 20-24 | T | 9.75 | 9.31 | 10.31 | 13.32 | 10.72 | 11.56 | 14.22 | 8.73 | 10.93 | 13.07 | 20.23 | 14.66 |
| | M | 14.06 | 11.37 | 10.77 | 18.26 | 13.52 | 16.98 | 17.33 | 10.55 | 14.69 | 15.99 | 26.48 | 18.44 |
| | F | 5.44 | 7.25 | 9.85 | 8.40 | 7.95 | 6.24 | 11.23 | 7.02 | 7.43 | 10.35 | 14.10 | 10.85 |
| 10-24 | T | 6.37 | 5.74 | 6.27 | 7.21 | 5.96 | 6.41 | 7.29 | 4.85 | 6.04 | 6.33 | 9.06 | 7.24 |
| | M | 8.14 | 6.17 | 6.22 | 9.47 | 6.65 | 8.73 | 8.76 | 5.25 | 7.17 | 7.54 | 10.99 | 8.37 |
| | F | 4.51 | 5.29 | 6.33 | 4.85 | 5.24 | 4.03 | 5.80 | 4.43 | 4.89 | 5.10 | 7.06 | 6.06 |

* T = (suicide deaths/mid year population) per 100,000 persons for the total population
M = (suicide deaths/mid year population) per 100,000 persons for the male population
F = (suicide deaths/mid year population) per 100,000 persons for the female population

Note. Sources: Census and Statistics Department, Hong Kong Special Administrative Region Government.

## TABLE 2

### Suicide rates for the 15-24 age bracket in different places (1990-1994)

| *Year/Places* | | France | U.K. | Australia | U.S.A. | Canada | Germany | Italy | Republic of Korea | Sweden | Japan | Singapore | China (Urban) | China (Rural) | China (Whole) | Hong* Kong | Taiwan |
|---|---|---|---|---|---|---|---|---|---|---|---|---|---|---|---|---|---|
| **1990** | Male | 14.1 | 12.2 | 26.6 | 22.0 | 24.6 | 14.4 | 5.9 | 10.7 | 14.7 | 9.2 | 13.1 | 6.1 | 16.2 | 10.7 | 7.4 | |
| | Female | 4.4 | 2.1 | 4.7 | 3.9 | 5.0 | 4.3 | 2.0 | 5.5 | 5.3 | 4.7 | 7.5 | 10.3 | 35.1 | 22.2 | 6.9 | |
| | Total | 9.3 | 7.3 | 15.9 | 13.2 | 15.0 | 9.5 | 4.0 | 8.1 | 10.1 | 7.0 | 10.4 | 8.1 | 25.5 | 16.2 | 7.1 | |
| **1991** | Male | 15.0 | 11.4 | 25.2 | 21.9 | 27.7 | 14.4 | 5.7 | 10.1 | 16.9 | 9.1 | 10.8 | | | | 7.3 | 7.8 |
| | Female | 4.1 | 2.3 | 6.2 | 3.8 | 4.0 | 3.6 | 1.6 | 4.8 | 5.0 | 4.7 | 7.2 | | | | 5.6 | 3.9 |
| | Total | 9.6 | 7.0 | 15.9 | 13.1 | 16.1 | 9.2 | 3.7 | 7.5 | 11.1 | 7.0 | 9.1 | | | | 6.5 | 5.9 |
| **1992** | Male | 14.0 | 12.2 | 27.3 | 21.9 | 24.7 | 13.0 | 6.5 | 10.8 | 10.0 | 10.2 | 9.2 | 5.6 | 17.4 | 11.4 | 9.7 | 5.3 |
| | Female | 4.3 | 2.3 | 5.6 | 3.7 | 6.0 | 3.3 | 2.0 | 5.3 | 6.7 | 4.7 | 4.1 | 10.6 | 36.7 | 23.9 | 6.6 | 2.5 |
| | Total | 9.2 | 7.4 | 16.7 | 12.9 | 15.5 | 8.3 | 4.3 | 8.1 | 8.4 | 7.5 | 6.7 | 7.9 | 26.9 | 17.4 | 8.2 | 4.3 |
| **1993** | Male | 18.2 | 12.6 | 23.7 | 22.4 | 23.8 | 12.7 | 7.1 | 11.8 | 12.0 | 10.1 | 8.5 | | | | 8.2 | 5.3 |
| | Female | 5.2 | 2.2 | 3.7 | 4.1 | 4.7 | 3.4 | 1.4 | 6.1 | 6.6 | 4.4 | 6.0 | | | | 4.4 | 2.5 |
| | Total | 11.8 | 7.5 | 13.9 | 13.5 | 14.4 | 8.2 | 4.3 | 9.1 | 9.4 | 7.3 | 7.2 | | | | 6.3 | 4.0 |
| **1994** | Male | 16.1 | 11.5 | 27.0 | 23.4 | 24.7 | 13.9 | 7.4 | 11.0 | 13.1 | 12.0 | 10.8 | 3.6 | 16.7 | 10.0 | 9.5 | 6.1 |
| | Female | 4.3 | 2.2 | 4.1 | 3.7 | 5.3 | 3.7 | 1.8 | 5.9 | 3.5 | 5.1 | 8.8 | 6.4 | 33.0 | 20.1 | 8.7 | 2.6 |
| | Total | 10.3 | 7.0 | 15.8 | 13.8 | 15.2 | 9.0 | 4.7 | 8.5 | 8.4 | 8.6 | 9.8 | 4.9 | 24.7 | 14.8 | 9.1 | 4.4 |

Note. Sources: World Health Statistics Annual [31,32,33,34,35,36,37,38]. Homepages of the World Health Organization (http://www.who.int/en/). Homepages of International Academy for Suicide Research (http://www.uni-wuerzburg.ed/IASR/suicide-table1.htm). Causes of Death-reports published by the Government of Australia (http://www.abs.gov.au/ausstats). National Vital Statistics Reports published by National Center for Health Statistics, United States of America (http://www.cdc.gov/nchs/). Statistisches Bundesamt of Germany (http://www.gbe-bund.de). National Statistical Office of the Republic of Korea (http://www.nso.go.kr). Department of Health of the Republic of China (http://www.doh.gov.tw).

* Source: Homepages of the World Health Organization (http://www.who.int/en/).

## TABLE 3

### Suicide rates for the 15-24 age bracket in different places (1995-2000)

| Year\Places | | France | U.K. | Australia | U.S.A. | Canada | Germany | Italy | Republic of Korea | Sweden | Japan | Singapore | China (Urban) | China (Rural) | China (Whole) | Hong* Kong | Taiwan |
|---|---|---|---|---|---|---|---|---|---|---|---|---|---|---|---|---|---|
| 1995 | Male | 15.2 | 11.0 | 23.1 | 22.5 | 24.7 | 13.3 | 7.3 | 11.5 | 13.4 | 11.3 | 10.4 | | | | 9.0 | 5.1 |
| | Female | 4.6 | 2.2 | 6.1 | 3.7 | 4.9 | 3.9 | 1.6 | 7.3 | 5.2 | 5.5 | 7.4 | | | | 5.6 | 2.7 |
| | Total | 10.0 | 6.7 | 14.8 | 13.3 | 15.0 | 8.7 | 4.5 | 9.5 | 9.4 | 8.4 | 8.9 | | | | 7.3 | 3.9 |
| 1996 | Male | 12.8 | 9.7 | 26.8 | 20.0 | 23.8 | 12.6 | 7.8 | 14.4 | 12.0 | 11.3 | 9.1 | | | | 11.4 | 5.5 |
| | Female | 4.2 | 2.5 | 4.5 | 3.6 | 4.6 | 3.5 | 1.7 | 9.0 | 4.6 | 5.4 | 2.8 | | | | 7.2 | 2.9 |
| | Total | 8.6 | 6.2 | 15.9 | 12.0 | 14.4 | 8.2 | 4.8 | 11.8 | 8.4 | 8.5 | 6.0 | | | | 9.3 | 4.2 |
| 1997 | Male | 13.4 | 11.1 | 30.4 | 18.9 | 22.4 | 12.9 | 8.5 | 12.5 | 11.6 | 11.3 | 6.9 | | | | 9.8 | |
| | Female | 4.3 | 2.3 | 6.7 | 3.5 | 4.5 | 3.2 | 1.8 | 8.0 | 6.6 | 5.5 | 4.7 | | | | 4.7 | |
| | Total | 8.9 | 6.8 | 18.7 | 11.4 | 13.7 | 8.2 | 5.2 | 10.3 | 9.1 | 8.5 | 5.8 | | | | 7.3 | 4.3 |
| 1998 | Male | 13.2 | 10.4 | 26.5 | 18.5 | 21.9 | 12.7 | 7.5 | 15.1 | 10.7 | 16.8 | 10.2 | 3.4 | 8.4 | | 13.9 | 5.1 |
| | Female | 3.6 | 2.9 | 6.3 | 3.3 | 5.3 | 3.5 | 1.9 | 9.4 | 5.5 | 7.4 | 7.2 | 4.4 | 15.2 | | 7.5 | 3.3 |
| | Total | 8.5 | 6.7 | 16.6 | 11.1 | 13.8 | 8.2 | 4.8 | 12.3 | 8.1 | 12.2 | 8.7 | | | | 10.7 | 4.2 |
| 1999 | Male | 12.3 | 10.7 | 22.8 | 17.2 | 22.2 | 12.7 | 6.8 | 12.2 | 14.8 | 16.5 | 7.0 | 3.0 | 8.0 | 5.4 | 9.0 | 5.6 |
| | Female | 3.4 | 2.5 | 5.5 | 3.1 | 4.5 | 3.0 | 1.7 | 7.9 | 6.4 | 7.3 | 3.9 | 4.1 | 12.9 | 8.6 | 6.8 | 3.1 |
| | Total | 7.9 | 6.7 | 14.3 | 10.3 | 13.5 | 8.0 | 4.3 | 10.1 | 10.7 | 12.0 | 5.5 | 3.5 | 10.4 | 6.9 | 7.9 | 4.3 |
| 2000 | Male | 12.1 | 10.5 | 20.3 | 17.0 | 20.2 | 12.2 | 6.7 | 10.2 | 12.0 | 15.8 | 6.5 | | | | 7.5 | |
| | Female | 3.6 | 2.9 | 6.1 | 3.0 | 5.5 | 3.0 | 1.6 | 7.0 | 5.2 | 6.9 | 7.7 | | | | 5.5 | 3.3 |
| | Total | 7.9 | 6.8 | 13.3 | 10.2 | 13.0 | 7.7 | 4.2 | 8.7 | 8.7 | 11.5 | 7.1 | | | | 6.5 | |

Note. Sources: World Health Statistics Annual [39]. Homepages of the World Health Organization (http://www.who.int/en/). Homepages of International Academy for Suicide Research (http://www.uni-wuerzburg.ed/IASR/suicide-table1.htm). Causes of Death-reports published by the Government of Australia (http://www.abs.gov.au/ausstats). National Vital Statistics Reports published by National Center for Health Statistics, United States of America (http://www.cdc.gov/nchs/). Statistisches Bundesamt of Germany (http://www.gbe-bund.de). National Statistical Office of the Republic of Korea (http://www.nso.go.kr). Department of Health of the Republic of China (http://www.doh.gov.tw).

* Source: Homepages of the World Health Organization (http://www.who.int/en/).

## TABLE 4

### Suicide rates for the 15-24 age bracket in different places (2001-2003)

| *Year\Places* | | France | U.K. | Australia | U.S.A. | Canada | Germany | Italy | Republic of Korea | Sweden | Japan | Singapore | China (Urban) | China (Rural) | China (Whole) | Hong* Kong | Taiwan |
|---|---|---|---|---|---|---|---|---|---|---|---|---|---|---|---|---|---|
| 2001 | Male | | 9.5 | 20.7 | 16.6 | | 12.4 | 6.6 | 9.2 | 11.2 | 14.6 | 9.7 | | | | 11.0 | 5.7 |
| | Female | | 1.8 | 4.8 | 2.9 | | 2.7 | 1.3 | 6.2 | 3.6 | 7.0 | 7.6 | | | | 6.8 | 3.7 |
| | Total | | 5.7 | 12.9 | 9.9 | | 7.7 | 4.0 | 7.7 | 7.5 | 10.9 | 8.7 | | | | 8.9 | 4.7 |
| 2002 | Male | | 8.2 | | 16.5 | | | | 9.7 | | 14.7 | | | | | 15.8 | 7.9 |
| | Female | | 2.4 | | 2.9 | | | | 7.1 | | 6.3 | | | | | 9.3 | 3.8 |
| | Total | | 5.3 | | 9.9 | | | | 8.4 | | 10.7 | | | | | 12.6 | 5.9 |
| 2003 | Male | | | | | | | | 12.9 | | | | | | | 12.6 | |
| | Female | | | | | | | | 8.9 | | | | | | | 8.3 | |
| | Total | | | | 9.5 | | | | 11.0 | | | | | | | 10.5 | |

Note. Sources: Homepages of the World Health Organization (http://www.who.int/en/). Homepages of International Academy for Suicide Research (http://www.uni-wuerzburg.ed/IASR/suicide-table1.htm). Causes of Death-reports published by the Government of Australia (http://www.abs.gov.au/ausstats). National Vital Statistics Reports published by National Center for Health Statistics, United States of America (http://www.cdc.gov/nchs/). Statistisches Bundesamt of Germany (http://www.gbe-bund.de). National Statistical Office of the Republic of Korea (http://www.nso.go.kr). Department of Health of the Republic of China (http://www.doh.gov.tw).

* Source: Census and Statistics Department, Hong Kong Special Administrative Region Government.

## TABLE 5

## Proportional mortality rates for suicide in Hong Kong (1980-2003)

| Age group | | 1980 | 1981 | 1982 | 1983 | 1984 | 1985 | 1986 | 1987 | 1988 | 1989 | 1990 | 1991 |
|---|---|---|---|---|---|---|---|---|---|---|---|---|---|
| 10-14 | *T | 1.71 | 4.51 | 0.00 | 3.64 | 1.14 | 3.23 | 5.00 | 0.00 | 3.70 | 0.00 | 3.57 | 8.54 |
| | *M | 0.00 | 4.29 | 0.00 | 1.82 | 0.00 | 1.85 | 5.77 | 0.00 | 4.00 | 0.00 | 2.27 | 9.09 |
| | *F | 4.35 | 4.76 | 0.00 | 5.45 | 2.56 | 5.13 | 4.17 | 0.00 | 3.23 | 0.00 | 5.00 | 7.89 |
| 15-19 | T | 12.18 | 6.64 | 9.18 | 8.33 | 6.49 | 12.32 | 11.49 | 9.84 | 10.39 | 11.27 | 10.56 | 14.06 |
| | M | 8.44 | 6.21 | 7.46 | 5.13 | 3.00 | 13.19 | 5.95 | 7.23 | 12.38 | 8.14 | 6.25 | 13.75 |
| | F | 19.05 | 7.69 | 12.33 | 13.33 | 12.96 | 10.64 | 18.75 | 15.38 | 6.12 | 16.07 | 16.92 | 14.58 |
| 20-24 | T | 15.62 | 15.48 | 9.64 | 10.37 | 15.49 | 25.30 | 23.98 | 19.23 | 21.17 | 16.33 | 19.46 | 21.57 |
| | M | 16.30 | 15.21 | 6.90 | 10.29 | 16.33 | 20.28 | 20.00 | 21.21 | 16.10 | 14.65 | 18.37 | 17.69 |
| | F | 14.15 | 15.97 | 16.00 | 10.49 | 13.64 | 33.70 | 32.10 | 15.79 | 26.92 | 19.15 | 21.62 | 28.38 |
| 10-24 | T | 12.06 | 10.50 | 7.87 | 8.63 | 10.46 | 17.29 | 16.40 | 12.81 | 14.44 | 12.21 | 13.52 | 16.67 |
| | M | 11.06 | 10.27 | 6.60 | 7.45 | 10.14 | 14.90 | 13.62 | 12.93 | 12.45 | 10.53 | 11.85 | 14.96 |
| | F | 13.98 | 10.93 | 11.42 | 10.26 | 11.05 | 21.35 | 20.73 | 12.59 | 17.39 | 14.84 | 16.20 | 19.38 |

| Age group | | 1992 | 1993 | 1994 | 1995 | 1996 | 1997 | 1998 | 1999 | 2000 | 2001 | 2002 | 2003 |
|---|---|---|---|---|---|---|---|---|---|---|---|---|---|
| 10-14 | T | 10.47 | 9.52 | 8.82 | 18.87 | 10.45 | 10.34 | 5.77 | 18.37 | 20.75 | 8.00 | 13.33 | 6.82 |
| | M | 11.11 | 5.13 | 2.56 | 20.83 | 9.38 | 12.82 | 3.03 | 12.90 | 15.15 | 3.85 | 7.89 | 0.00 |
| | F | 9.38 | 16.67 | 17.24 | 17.24 | 11.43 | 5.26 | 10.53 | 27.78 | 30.00 | 12.50 | 22.73 | 14.29 |
| 15-19 | T | 20.42 | 17.11 | 21.48 | 18.90 | 15.13 | 20.80 | 25.44 | 16.84 | 24.69 | 21.28 | 23.08 | 31.03 |
| | M | 17.98 | 14.94 | 19.51 | 19.77 | 11.11 | 18.52 | 25.68 | 13.56 | 24.44 | 22.95 | 21.05 | 26.79 |
| | F | 24.53 | 20.00 | 24.53 | 17.07 | 22.64 | 25.00 | 25.00 | 22.22 | 25.00 | 18.18 | 26.47 | 38.71 |
| 20-24 | T | 21.50 | 20.71 | 21.70 | 26.79 | 21.61 | 22.27 | 33.17 | 21.81 | 32.69 | 36.97 | 44.17 | 36.72 |
| | M | 21.38 | 18.94 | 16.33 | 25.63 | 20.25 | 21.16 | 29.85 | 19.83 | 29.73 | 32.14 | 43.07 | 34.17 |
| | F | 21.82 | 24.24 | 33.85 | 29.69 | 24.36 | 25.86 | 39.71 | 25.37 | 40.00 | 47.17 | 46.38 | 42.11 |
| 10-24 | T | 18.93 | 17.68 | 19.52 | 23.27 | 17.80 | 20.23 | 26.90 | 19.88 | 28.28 | 27.51 | 33.61 | 20.84 |
| | M | 18.40 | 15.50 | 15.30 | 23.33 | 15.92 | 19.42 | 24.90 | 17.06 | 25.93 | 25.63 | 31.90 | 28.14 |
| | F | 20.00 | 21.29 | 27.21 | 23.13 | 21.08 | 22.31 | 30.71 | 24.79 | 32.67 | 30.91 | 36.80 | 35.78 |

Note. Sources: Census and Statistics Department, Hong Kong Special Administrative Region Government.

**TABLE 6**

**Proportional mortality rates for suicide for the 15-24 age group in different places (1987-1995)**

| ***Year\Place*** | | **Hong * Kong** | **China (Whole)** | **China (Urban)** | **China (Rural)** | **U.S.A.** | **U.K.** | **Japan** | **Singapore** |
|---|---|---|---|---|---|---|---|---|---|
| **1987** | Male | 16.2 | 17.0 | 10.7 | 21.6 | 14.6 | 12.3 | 16.8 | 10.5 |
| | Female | 18.3 | 38.9 | 27.7 | 45.2 | 8.3 | 7.8 | 24.1 | 17.0 |
| | Total | 17.0 | 27.0 | 17.8 | 33.0 | 13.0 | 11.1 | 18.8 | 12.6 |
| **1988** | Male | 14.2 | 17.2 | 11.1 | 20.7 | 14.5 | 15.3 | 14.4 | 14.1 |
| | Female | 23.4 | 36.2 | 25.0 | 41.1 | 8.2 | 8.8 | 23.9 | 26.6 |
| | Total | 17.8 | 25.9 | 16.9 | 30.6 | 12.9 | 13.6 | 16.9 | 17.8 |
| **1989** | Male | 14.2 | 16.5 | 10.0 | 21.1 | 15.1 | 13.2 | 13.7 | 15.2 |
| | Female | 17.7 | 38.3 | 26.3 | 44.3 | 8.2 | 6.4 | 19.8 | 32.1 |
| | Total | 15.5 | 26.5 | 16.6 | 32.4 | 13.4 | 11.3 | 15.3 | 21.0 |
| **1990** | Male | 12.9 | 13.8 | 9.6 | 17.1 | 14.9 | 14.3 | 13.1 | 15.3 |
| | Female | 19.0 | 33.8 | 23.5 | 39.4 | 8.0 | 6.9 | 17.5 | 21.0 |
| | Total | 15.2 | 22.6 | 14.9 | 27.8 | 13.3 | 12.5 | 14.3 | 16.9 |
| **1991** | Male | 15.1 | | | | 14.8 | 13.9 | 13.5 | 17.2 |
| | Female | 19.8 | | | | 7.6 | 7.3 | 19.0 | 20.3 |
| | Total | 16.8 | | | | 13.0 | 12.1 | 15.0 | 18.3 |
| **1992** | Male | 19.9 | 14.9 | 9.7 | 18.1 | 15.4 | 16.3 | 15.2 | 14.1 |
| | Female | 24.6 | 37.2 | 25.4 | 42.6 | 7.8 | 7.6 | 18.2 | 14.5 |
| | Total | 21.5 | 24.6 | 15.8 | 29.4 | 13.6 | 13.8 | 16.0 | 14.2 |
| **1993** | Male | 15.1 | | | | 15.3 | 16.8 | 15.7 | 13.0 |
| | Female | 15.2 | | | | 8.5 | 7.3 | 18.1 | 24.1 |
| | Total | 15.2 | | | | 13.7 | 14.2 | 16.4 | 16.0 |
| **1994** | Male | 19.9 | 13.6 | 6.8 | 17.5 | 16.1 | 15.5 | 19.0 | 16.9 |
| | Female | 31.7 | 35.0 | 19.4 | 41.2 | 7.7 | 7.9 | 20.7 | 26.8 |
| | Total | 24.2 | 22.6 | 11.3 | 28.1 | 14.1 | 13.5 | 19.5 | 20.2 |
| **1995** | Male | 16.8 | | | | 16.0 | 14.3 | 17.4 | 17.0 |
| | Female | 23.3 | | | | 7.7 | 7.8 | 20.4 | 28.6 |
| | Total | 18.8 | | | | 14.0 | 12.6 | 18.2 | 20.4 |

Note. Sources: World Health Statistics Annual [31,32,33,34,35,36,37,38]. Homepages of the World Health Organization (http://www.who.int/en/).

*Source: Homepages of the World Health Organization (http://www.who.int/en/).

## TABLE 7

### Proportional mortality rates for suicide for the 15-24 age group in different places (1996-2003)

| *Year\Place* | | Hong Kong * | China (Whole) | China (Urban) | China (Rural) | U.S.A. | U.K. | Japan | Singapore |
|---|---|---|---|---|---|---|---|---|---|
| **1996** | Male | 21.9 | | | | 15.3 | 12.9 | 19.1 | 14.9 |
| | Female | 33.3 | | | | 7.8 | 8.1 | 22.7 | 10.2 |
| | Total | 25.2 | | | | 13.4 | 11.6 | 20.1 | 13.5 |
| **1997** | Male | 15.8 | | | | 15.3 | 14.6 | 19.7 | 11.3 |
| | Female | 17.3 | | | | 7.6 | 7.7 | 23.4 | 17.9 |
| | Total | 16.2 | | | | 13.3 | 12.7 | 20.7 | 13.2 |
| **1998** | Male | 28.4 | | | | 15.6 | 14.7 | 27.4 | 21.0 |
| | Female | 35.0 | | | | 7.6 | 9.6 | 29.0 | 28.3 |
| | Total | 30.4 | | | | 13.5 | 13.2 | 27.9 | 23.4 |
| **1999** | Male | 21.2 | | | | 14.8 | 14.9 | 27.9 | 13.4 |
| | Female | 28.1 | | | | 7.0 | 8.6 | 28.8 | 14.8 |
| | Total | 23.7 | | | | 12.7 | 13.1 | 28.2 | 13.9 |
| **2000** | Male | 21.8 | | | | 14.8 | 15.4 | 27.7 | 12.6 |
| | Female | 29.7 | | | | 6.9 | 10.3 | 29.0 | 32.0 |
| | Total | 24.5 | | | | 12.7 | 14.0 | 28.1 | 18.6 |
| **2001** | Male | 28.9 | | | | 14.2 | 13.5 | 26.8 | 16.7 |
| | Female | 36.1 | | | | 6.8 | 6.7 | 30.5 | 29.6 |
| | Total | 31.3 | | | | 12.3 | 11.6 | 27.9 | 20.6 |
| **2002** | Male | 36.6 | | | | 14.1 | 12.2 | 28.2 | |
| | Female | 39.8 | | | | 6.7 | 9.1 | 27.9 | |
| | Total | 37.7 | | | | 12.1 | 11.4 | 28.1 | |
| **2003** | Male | 31.8 | | | | | | | |
| | Female | 40.9 | | | | | | | |
| | Total | 34.9 | | | | 11.9 | | | |

Note. Sources: World Health Statistics Annual [31,32,33,34,35,36,37,38]. Homepages of the World Health Organization (http://www.who.int/en/).

*Sources: Hong Kong figures in 1996-2000 are from Homepages of the World Health Organization (http://www.who.int/en/). Hong Kong figures in 2001-2003 are from Census and Statistics Department, Hong Kong Special Administrative Region Government.

**TABLE 8**
**Methods of adolescent suicide in the 10-19 and 20-29 age groups in percentages (1989-1994)**

| Year | Age group | | Firearms | Drugs | Poisons | Hanging | Jumping from a height | Carbon monoxide | Drowning | Sharp instruments | Others |
|---|---|---|---|---|---|---|---|---|---|---|---|
| 1989 | 10-19 | T * | 0.0 | 0.0 | 0.0 | 13.3 | 80.0 | 0.0 | 0.0 | 0.0 | 6.7 |
| | | M * | 0.0 | 0.0 | 0.0 | 25.0 | 62.5 | 0.0 | 0.0 | 0.0 | 12.5 |
| | | F * | 0.0 | 0.0 | 0.0 | 0.0 | 100.0 | 0.0 | 0.0 | 0.0 | 0.0 |
| | 20-29 | T | 3.2 | 0.0 | 4.2 | 24.2 | 60.0 | 2.1 | 2.1 | 1.1 | 3.2 |
| | | M | 5.3 | 0.0 | 3.5 | 26.3 | 56.1 | 3.5 | 1.8 | 1.8 | 1.8 |
| | | F | 0.0 | 0.0 | 5.3 | 21.1 | 65.8 | 0.0 | 2.6 | 0.0 | 5.3 |
| 1990 | 10-19 | T | 0.0 | 0.0 | 25.0 | 0.0 | 75.0 | 0.0 | 0.0 | 0.0 | 0.0 |
| | | M | 0.0 | 0.0 | 100.0 | 0.0 | 0.0 | 0.0 | 0.0 | 0.0 | 0.0 |
| | | F | 0.0 | 0.0 | 0.0 | 0.0 | 100.0 | 0.0 | 0.0 | 0.0 | 0.0 |
| | 20-29 | T | 0.0 | 0.0 | 8.3 | 19.4 | 66.7 | 0.0 | 2.8 | 0.0 | 2.8 |
| | | M | 0.0 | 0.0 | 15.0 | 30.0 | 50.0 | 0.0 | 0.0 | 0.0 | 5.0 |
| | | F | 0.0 | 0.0 | 0.0 | 6.3 | 87.5 | 0.0 | 6.3 | 0.0 | 0.0 |
| 1991 | 10-19 | T | 0.0 | 0.0 | 0.0 | 5.3 | 89.5 | 0.0 | 0.0 | 5.3 | 0.0 |
| | | M | 0.0 | 0.0 | 0.0 | 7.7 | 84.6 | 0.0 | 0.0 | 7.7 | 0.0 |
| | | F | 0.0 | 0.0 | 0.0 | 0.0 | 100.0 | 0.0 | 0.0 | 0.0 | 0.0 |
| | 20-29 | T | 0.0 | 3.3 | 0.0 | 16.7 | 66.7 | 4.2 | 3.3 | 2.5 | 3.3 |
| | | M | 0.0 | 2.8 | 0.0 | 15.5 | 66.2 | 4.2 | 4.2 | 2.8 | 4.2 |
| | | F | 0.0 | 4.1 | 0.0 | 18.4 | 67.3 | 4.1 | 2.0 | 2.0 | 2.0 |
| 1992 | 10-19 | T | 0.0 | 0.0 | 0.0 | 5.3 | 89.5 | 0.0 | 0.0 | 5.3 | 0.0 |
| | | M | 0.0 | 5.0 | 0.0 | 5.0 | 90.0 | 0.0 | 0.0 | 0.0 | 0.0 |
| | | F | 0.0 | 0.0 | 5.6 | 5.8 | 83.3 | 0.0 | 5.6 | 0.0 | 0.0 |
| | 20-29 | T | 0.0 | 3.3 | 0.0 | 16.7 | 66.7 | 4.2 | 3.3 | 2.5 | 3.3 |
| | | M | 1.5 | 0.0 | 3.1 | 15.4 | 67.7 | 4.6 | 3.1 | 0.0 | 4.6 |
| | | F | 0.0 | 2.0 | 4.1 | 12.2 | 69.4 | 4.1 | 4.1 | 0.0 | 4.1 |
| 1993 | 10-19 | T | 0.0 | 3.6 | 0.0 | 0.0 | 85.7 | 0.0 | 7.1 | 0.0 | 3.6 |
| | | M | 0.0 | 6.7 | 0.0 | 0.0 | 86.7 | 0.0 | 0.0 | 0.0 | 6.7 |
| | | F | 0.0 | 0.0 | 0.0 | 0.0 | 84.5 | 0.0 | 15.4 | 0.0 | 0.0 |
| | 20-29 | T | 2.0 | 4.1 | 4.1 | 17.3 | 60.2 | 3.1 | 2.0 | 0.0 | 7.1 |
| | | M | 3.0 | 4.5 | 6.1 | 21.2 | 56.1 | 1.5 | 3.0 | 0.0 | 4.5 |
| | | F | 0.0 | 3.1 | 0.0 | 9.4 | 68.8 | 6.3 | 0.0 | 0.0 | 12.5 |
| 1994 | 10-19 | T | 0.0 | 0.0 | 2.7 | 0.0 | 94.6 | 0.0 | 0.0 | 0.0 | 2.7 |
| | | M | 0.0 | 0.0 | 0.0 | 0.0 | 100.0 | 0.0 | 0.0 | 0.0 | 0.0 |
| | | F | 0.0 | 0.0 | 5.3 | 0.0 | 89.5 | 0.0 | 0.0 | 0.0 | 5.3 |
| | 20-29 | T | 0.8 | 1.7 | 3.4 | 13.6 | 71.2 | 0.8 | 1.7 | 0.8 | 5.9 |
| | | M | 1.5 | 1.5 | 3.0 | 13.6 | 68.2 | 1.5 | 0.0 | 1.5 | 9.1 |
| | | F | 0.0 | 1.9 | 3.8 | 13.5 | 75.0 | 0.0 | 3.8 | 0.0 | 1.9 |

* T = Total population
M = Male population
F = Female population

Note. Sources: Coroners Report [40,41,42,43,44,45].

**TABLE 9**
**Methods of adolescent suicide in the 10-19 and 20-29 age groups in percentages (1995-2000)**

| Year | Age group | | Firearms | Drugs | Poisons | Hanging | Jumping from a height | Carbon monoxide | Drowning | Sharp instruments | Others |
|---|---|---|---|---|---|---|---|---|---|---|---|
| 1995 | 10-19 | T * | 0.0 | 0.0 | 0.0 | 3.6 | 92.9 | 0.0 | 3.6 | 0.0 | 0.0 |
| | | M * | 0.0 | 0.0 | 0.0 | 5.9 | 88.2 | 0.0 | 5.9 | 0.0 | 0.0 |
| | | F * | 0.0 | 0.0 | 0.0 | 0.0 | 100.0 | 0.0 | 0.0 | 0.0 | 0.0 |
| | 20-29 | T | 0.0 | 2.8 | 1.8 | 12.8 | 75.2 | 1.8 | 1.8 | 0.0 | 3.7 |
| | | M | 0.0 | 3.0 | 1.5 | 13.4 | 73.1 | 3.0 | 3.0 | 0.0 | 3.0 |
| | | F | 0.0 | 2.4 | 2.4 | 11.9 | 78.6 | 0.0 | 0.0 | 0.0 | 4.8 |
| 1996 | 10-19 | T | 0.0 | 2.7 | 2.7 | 10.8 | 81.1 | 0.0 | 0.0 | 0.0 | 2.7 |
| | | M | 0.0 | 0.0 | 0.0 | 13.6 | 81.8 | 0.0 | 0.0 | 0.0 | 4.5 |
| | | F | 0.0 | 6.7 | 6.7 | 6.7 | 80.0 | 0.0 | 0.0 | 0.0 | 0.0 |
| | 20-29 | T | 1.6 | 1.6 | 3.9 | 10.2 | 73.4 | 2.3 | 3.9 | 0.0 | 3.1 |
| | | M | 2.5 | 1.2 | 4.9 | 11.1 | 67.9 | 2.5 | 4.9 | 0.0 | 4.9 |
| | | F | 0.0 | 2.1 | 2.1 | 8.5 | 83.0 | 2.1 | 2.1 | 0.0 | 0.0 |
| 1997 | 10-19 | T | 0.0 | 3.7 | 3.7 | 11.1 | 77.8 | 0.0 | 3.7 | 0.0 | 0.0 |
| | | M | 0.0 | 7.7 | 7.7 | 23.1 | 61.5 | 0.0 | 0.0 | 0.0 | 0.0 |
| | | F | 0.0 | 0.0 | 0.0 | 0.0 | 92.8 | 0.0 | 7.1 | 0.0 | 0.0 |
| | 20-29 | T | 0.0 | 8.9 | 0.0 | 21.1 | 61.1 | 4.4 | 0.0 | 1.1 | 3.3 |
| | | M | 0.0 | 10.3 | 0.0 | 25.0 | 57.4 | 4.4 | 0.0 | 1.5 | 1.5 |
| | | F | 0.0 | 4.5 | 0.0 | 9.1 | 72.7 | 4.5 | 0.0 | 0.0 | 9.1 |
| 1998 | 10-19 | T | 0.0 | 0.0 | 0.0 | 30.6 | 69.4 | 0.0 | 0.0 | 0.0 | 0.0 |
| | | M | 0.0 | 0.0 | 0.0 | 43.5 | 56.5 | 0.0 | 0.0 | 0.0 | 0.0 |
| | | F | 0.0 | 0.0 | 0.0 | 7.7 | 92.3 | 0.0 | 0.0 | 0.0 | 0.0 |
| | 20-29 | T | 0.6 | 2.5 | 0.0 | 19.3 | 67.7 | 3.1 | 4.3 | 0.0 | 2.5 |
| | | M | 1.0 | 2.9 | 0.0 | 20.2 | 64.4 | 3.8 | 3.8 | 0.0 | 3.8 |
| | | F | 0.0 | 1.8 | 0.0 | 17.5 | 73.9 | 1.8 | 5.3 | 0.0 | 0.0 |
| 1999 | 10-19 | T | 0.0 | 4.0 | 0.0 | 12.0 | 80.0 | 4.0 | 0.0 | 0.0 | 0.0 |
| | | M | 0.0 | 0.0 | 0.0 | 15.4 | 76.9 | 7.7 | 0.0 | 0.0 | 0.0 |
| | | F | 0.0 | 8.3 | 0.0 | 8.3 | 83.3 | 0.0 | 0.0 | 0.0 | 0.0 |
| | 20-29 | T | 0.0 | 3.7 | 0.9 | 16.7 | 60.2 | 11.1 | 2.8 | 0.0 | 4.6 |
| | | M | 0.0 | 1.5 | 0.0 | 22.7 | 54.5 | 13.6 | 1.5 | 0.0 | 6.1 |
| | | F | 0.0 | 7.1 | 2.4 | 7.1 | 69.0 | 7.1 | 4.8 | 0.0 | 2.4 |
| 2000 | 10-19 | T | 0.0 | 3.8 | 0.0 | 3.8 | 88.5 | 0.0 | 3.8 | 0.0 | 0.0 |
| | | M | 0.0 | 0.0 | 0.0 | 7.1 | 85.7 | 0.0 | 7.1 | 0.0 | 0.0 |
| | | F | 0.0 | 8.3 | 0.0 | 0.0 | 91.7 | 0.0 | 0.0 | 0.0 | 0.0 |
| | 20-29 | T | 0.0 | 2.9 | 0.0 | 11.7 | 54.0 | 24.8 | 2.2 | 0.7 | 1.0 |
| | | M | 0.0 | 1.2 | 0.0 | 17.4 | 47.7 | 26.7 | 0.0 | 1.2 | 5.8 |
| | | F | 0.0 | 5.9 | 0.0 | 2.0 | 64.7 | 21.6 | 5.9 | 0.0 | 0.0 |

* T = Total population
M = Male population
F = Female population

Note. Sources: Coroners Report [46,47,48,49,50,51]

## TABLE 10

### Methods of adolescent suicide in the 10-19 and 20-29 age groups in percentages (2001-2003)

| Year | Age group | | Firearms | Drugs | Poisons | Hanging | Jumping from a height | Carbon monoxide | Drowning | Sharp instruments | Others |
|---|---|---|---|---|---|---|---|---|---|---|---|
| 2001 | 10-19 | T * | 0.0 | 0.0 | 0.0 | 3.1 | 84.4 | 12.5 | 0.0 | 0.0 | 0.0 |
| | | M * | 0.0 | 0.0 | 0.0 | 5.9 | 88.2 | 5.9 | 0.0 | 0.0 | 0.0 |
| | | F * | 0.0 | 0.0 | 0.0 | 0.0 | 80.0 | 20.0 | 0.0 | 0.0 | 0.0 |
| | 20-29 | T | 0.6 | 5.9 | 0.0 | 7.0 | 45.6 | 38.0 | 1.8 | 0.0 | 1.2 |
| | | M | 0.9 | 7.0 | 0.0 | 8.8 | 46.5 | 36.0 | 0.0 | 0.0 | 0.9 |
| | | F | 0.0 | 3.5 | 0.0 | 3.5 | 43.9 | 42.1 | 5.3 | 0.0 | 1.8 |
| 2002 | 10-19 | T | 0.0 | 3.8 | 0.0 | 26.9 | 69.2 | 0.0 | 0.0 | 0.0 | 0.0 |
| | | M | 0.0 | 0.0 | 0.0 | 26.7 | 73.3 | 0.0 | 0.0 | 0.0 | 0.0 |
| | | F | 0.0 | 9.1 | 0.0 | 27.3 | 63.6 | 0.0 | 0.0 | 0.0 | 0.0 |
| | 20-29 | T | 0.0 | 2.7 | 0.5 | 10.8 | 57.0 | 25.3 | 3.2 | 0.5 | 0.0 |
| | | M | 0.0 | 2.5 | 0.0 | 11.9 | 57.6 | 24.6 | 3.4 | 0.0 | 0.0 |
| | | F | 0.0 | 2.9 | 1.5 | 8.8 | 55.9 | 26.5 | 2.9 | 1.5 | 0.0 |
| 2003 | 10-19 | T | 0.0 | 0.0 | 0.0 | 6.9 | 72.4 | 20.7 | 0.0 | 0.0 | 0.0 |
| | | M | 0.0 | 0.0 | 0.0 | 15.4 | 61.5 | 23.1 | 0.0 | 0.0 | 0.0 |
| | | F | 0.0 | 0.0 | 0.0 | 0.0 | 81.3 | 18.8 | 0.0 | 0.0 | 0.0 |
| | 20-29 | T | 0.0 | 1.8 | 1.2 | 9.8 | 51.8 | 33.5 | 1.2 | 0.0 | 0.6 |
| | | M | 0.0 | 0.0 | 1.7 | 7.7 | 53.9 | 35.0 | 0.9 | 0.0 | 0.9 |
| | | F | 0.0 | 6.4 | 0.0 | 14.9 | 46.8 | 29.8 | 2.1 | 0.0 | 0.0 |

* T = Total population
M = Male population
F = Female population

Note. Sources: Coroners Report [52,53,54].
